# Oropharyngeal tumor cells induce COX-2 expression in peripheral blood monocytes by secretion of IL-1α

**DOI:** 10.3389/fimmu.2022.1011772

**Published:** 2022-11-08

**Authors:** James A. DeVoti, Mohd Israr, Fung Lam, Christopher Papayannakos, Douglas K. Frank, Dev P. Kamdar, Lucio M. Pereira, Allan Abramson, Bettie M. Steinberg, Vincent R. Bonagura

**Affiliations:** ^1^ Institute of Molecular Medicine, Feinstein Institutes for Medical Research, Manhasset, NY, United States; ^2^ Department of Pediatrics, Steven and Alexandra Cohen Children’s Medical Center of New York, Barbara and Donald Zucker School of Medicine at Hofstra/Northwell, Hempstead, NY, United States; ^3^ Department of Otolaryngology, Long Island Jewish Medical Center, Barbara and Donald Zucker School of Medicine at Hofstra/Northwell, Hempstead, NY, United States; ^4^ Department of Molecular Medicine, Barbara and Donald Zucker School of Medicine at Hofstra/Northwell, Hempstead, NY, United States

**Keywords:** PGE 2, IL-1 alpha, COX-2, oropharyngeal cancer, monocytes

## Abstract

Oropharyngeal squamous cell cancer (OPC) accounts for 3% of all cancers and greater than 1.5% of all cancer deaths in the United States, with marked treatment-associated morbidity in survivors. More than 80% of OPC is caused by HPV16. Tumors induced by HPV have been linked to impaired immune functions, with most studies focused on the local tumor microenvironment. Fewer studies have characterized the effects of these tumors on systemic responses in OPC, especially innate responses that drive subsequent adaptive responses, potentially creating feed-back loops favorable to the tumor. Here we report that elevated plasma levels of PGE_2_ are expressed in half of patients with OPC secondary to overexpression of COX-2 by peripheral blood monocytes, and this expression is driven by IL-1α secreted by the tumors. Monocytes from patients are much more sensitive to the stimulation than monocytes from controls, suggesting the possibility of enhanced immune-modulating feed-back loops. Furthermore, control monocytes pre-exposed to PGE_2_ overexpress COX-2 in response to IL-1α, simulating responses made by monocytes from some OPC patients. Disrupting the PGE_2_/IL-1α feed-back loop can have potential impact on targeted medical therapies.

## Introduction

Head and neck cancer, which includes oropharyngeal cancer of the palatine tonsil and base of tongue (OPC), is the 6^th^ or 7^th^ most common cancer worldwide, with between 6 and 9 x10^5^ new cases/year and approximately 4.5 x 10^5^ deaths annually (1, 2). In the United States, OPC accounts for 3% of all cancers and greater than 1.5% of all cancer deaths (2). More than 80% of OPC is caused by HPV16 (1) with smoking and alcohol abuse the main risk factors for non-HPV induced OPC.

The development of tumors induced by HPVs has been linked to the failure of effector HPV-specific immune competence, either because of a genetic defect, co-infection with other viruses, such as HIV, or biased immune responses likely related to expression of immune modulators that impair innate and adaptive effector function (3–9). A recent retrospective study showed that the use of non-steroidal anti-inflammatory drugs correlated with better clinical outcomes in a third of HPV-positive OPC patients with *PI3KCA* activating mutations or gene duplications (8). That data suggests that HPV^+^ patients could potentially be subdivided into two subgroups for therapeutic considerations. Here, we provide evidence for additional subgroups, based on elevated plasma PGE_2_ levels independent of *PI3KCA* mutations. Defining sub-categories for individual therapy is needed, given that outcomes for patients with HPV^+^ tumors are generally better than those who have HPV-negative tumors (10). However, standard treatment is largely the same for both groups despite the significant long-term morbidity of current treatment protocols and decreasing standard therapy for HPV16-positive OPC patients appears to be detrimental to their overall clinical outcome (2, 11).

PGE_2_ can significantly alter innate and adaptive immune function (12), and those altered responses are characteristic of some HPV^+^ OPC patients (13, 14). Serum PGE_2_ was elevated in mice who received cutaneous explants from human tumors with enhanced *PI3KCA* activity (8). Most recent tumor immunology studies focus on the local tumor environment including the tumor cells themselves, tumor-associated macrophages, dendritic cells, cancer-associated fibroblasts, and tumor-infiltrating lymphocytes. Fewer studies have characterized the effects of these OPC tumors on systemic responses, especially innate responses that bias signaling by monocyte and dendritic cells, and the potential impact of these alterations on tumor survival and progression (15). We have previously shown a systemic “immunopathy” in persistent HPV6/11 infections of the larynx and upper airway in patients with recurrent respiratory papillomas (RRP), suggesting a possible common mechanism (16–19).

In this communication, we describe the elevation of PGE_2_ in the plasma of a large subgroup of HPV16^+^ OPC patients. We show that a subset of OPC tumors and tumor cell lines secrete IL-1α that drives elevated gene expression of *PTGS2 (COX-2)* by monocytes isolated from circulating PBMC, independent of alterations in *PIK3CA*. We also show that patient’s monocytes are hyper-responsive to stimulation by IL-1α present in conditioned media, and that control monocytes show hyper-responsiveness by combined PGE_2_ and IL-1α exposure. Defining systemic innate alterations in OPC patients can help further characterize subpopulations of OPC patients, based on their innate immunopathology, that may have impact on personalized, targeted medical therapies.

## Materials and methods

### Human subjects

This study used biopsies of OPC tumors and contralateral, clinically normal epithelium (hereafter called “normal adjacent” tissue), peripheral blood samples from OPC patients and controls without OPC, and normal tonsil surgical discards from patients with obstructive sleep apnea. All samples were obtained from patients at Long Island Jewish Medical Center, Northwell Health. This study was approved by the Northwell Health Institutional Review Board. Biopsies were obtained within one hour post-surgery and cultured or snap frozen for subsequent analysis. Blood samples were processed immediately as described below.

### Measurement of prostaglandin E_2_ plasma levels

Heparinized whole blood was centrifuged at 1200x g for ten minutes at room temperature. Plasma aliquots were immediately frozen at -80°C until use. A forward sequential competitive binding ELISA (PKGE004B, R&D Systems) was used as per the manufactures specifications to detect plasma PGE_2_ expression. The cross reaction of anti-PGE_2_ antibodies with other prostanoids is <5% as per the manufacturer. All samples were run in triplicate, and mean plasma PGE_2_ levels calculated for each sample.

### Identification of *PIK3CA* activating mutations and/or gene duplication in OPCs

Genomic DNA and total RNA were isolated simultaneously from matched sets of tumor and clinically normal tonsil/base of tongue biopsies from each OPC patient using the AllPrep DNA/RNA Mini Kit (Qiagen, Hilden, Germany) as per manufacturer’s instructions. Copy number analysis of the *PIK3CA* gene was performed on genomic DNA using Taqman Copy Number Assays (Applied Biosystems) on the 7900 H.T. Fast Real Time PCR system and analyzed with the *CopyCaller Software v2.0* as per manufacturer’s instructions. *PIK3CA* specific primers and probe, Hs04769644_cn, was run together with 2 individual copy number reference assays, RNase P (4401631), and H-TERT (4401633) using the Taq-Man Geneotyping Master Mix (4371355) for 1 cycle at 95°C X 10min. (denaturation and hot-start) followed by 40 cycles of 95°C for 15 sec. (denature) and 60°C for 60 sec. (anneal/extend) as per manufacturer’s specifications. Total RNA from each matched set of biopsies was amplified with primers that gave a single RT-PCR product containing both the 542 and the 545 amino acid positions (sense primer: cccagatatgtcagtgattg, antisense primer: ccaagcaatacatctgggctac) or with primers that gave a single RT-PCR product that contained both the 1047 and the 1048 amino acid positions (sense primer: tgcacaaagacaagagaatttga, antisense primer: gcaattcctatgcaatcggtct). The PCR products were run on a 2% Low-melt agarose gel (Sea Plaque, BioWhittaker Molecular Applications, Rockland, ME.), gel purified with the MinElute Gel Extraction Kit (Qiagen) and quantitated by spectrophotometry. Twenty ng. of gel-purified PCR product and 5 uM of either primer was used for sequencing the amplicon in both directions by the Sanger method, performed by a commercial lab (Genewiz, South Plainfield, NJ).

### Isolation of total PBMC and monocytes

To isolate PBMC, 20 mL of heparinized blood was diluted to 30 mL with RPMI supplemented with 100 μnits/mL penicillin, 100 μg/mL streptomycin and 2mM L-glutamine, layered over Ficoll-Paque Plus (GE Healthcare, Uppsala, Sweden), and then centrifuged for 20 min. at room temp at 500 x g. The lymphocyte layer was collected and washed twice with RPMI1640 culture medium (Life Technologies Limited, Paisley, U.K.). Monocytes were isolated by negative selection using the Pan Monocyte Isolation Kit (Miltenyi Biotec, Bergish Gladbach, Germany), as per the manufacturer’s instructions. Only purified monocytes with a yield of CD14^+^ cells greater than 90% were used in subsequent experiments.

### Q-RT-PCR measurement of COX-2 mRNA

Total RNA was isolated using the RNeasy Mini Kit (Qiagen, Hilden, Germany) as per manufacturer’s instructions and digested with the RNase-Free DNase-1 Set (Qiagen, Hilden, Germany) to remove contaminating genomic DNA. Reverse transcription and amplification was performed with the iTaq Universal Probes One-step Kit (BIO-RAD, Hercules, CA) as per manufacturer’s instructions. TaqMan gene expression assays comprised of intron-spanning primers and gene specific probes were purchased from Applied Bio-systems. A GAPDH-specific assay, Hs99999905_m1, was used to measure mRNA expression of this housekeeping gene in individual samples for comparison to *PTGS2*, Hs00153133_m1 (*COX-2*) mRNA expression in total RNA samples using the ddCt method. All samples were run in triplicate on the Applied Bio-systems 7900 H.T Fast Real Time PCR System.

### Culture of biopsy tissues and cell lines

Tissues were washed thoroughly in PBS containing 100 μg/mL streptomycin and 100 U/mL of penicillin, cut into small fragments, and incubated with 0.05% trypsin (Gibco, Grand Island, NY, USA) for 30 min at 37°C. Cells were plated on J2-3T3 feeder cells in 60 mm culture plates in E-medium (DMEM (Gibco, Grand Island, NY)/Ham’s F-12 (Gibco, Grand Island, NY) at a 3:1 ratio, supplemented with 10% Fetal Clone II (Hyclone, Logan, UT), 0.4 μg/mL hydrocortisone, 0.1 nM cholera toxin, 5 μg/mL transferrin, 2 nM 3,3-5-triodo-L-thyronine, 5ng/mL EGF, 5μg/mL insulin, (Sigma, Saint Louis, MO), and 100 units/mL penicillin and 100 μg/mL streptomycin (Gibco, Grand Island, NY). Cultures were maintained at 37°C in 5% CO_2_-enriched air, and sub-cultured when they reached 70–80% confluence. Tumor cells were used within five passages. Oral/oropharyngeal cancer cell lines SCC-4, SCC-25, SCC-090, SCC-152 and SCC-154 were purchased from the American Type Culture Collection (ATCC) (Manassas, VA) and maintained in supplemented E-medium as above.

### Preparation of conditioned media

Keratinocytes from OPC tumors, normal adjacent tissue, normal tonsil, or the cell lines were cultured as described above, the medium replaced with fresh E-medium containing 2% Fetal Clone II and 100 units/mL penicillin and 100μg/mL streptomycin. Conditioned medium was collected after 48 hrs, centrifuged at 4000x g for 20 min, passed through a 0.22um filter to remove any cellular debris, and used immediately to stimulate monocytes or stored at −80°C for later experiments. PGE_2_ levels in conditioned medium were measured by ELISA as described above.

### Stimulation of monocytes

Isolated monocytes at 2.5 x 10^5^ cells/well were cultured in 24 well plates (Falcon #353047, Corning, New York) in E-medium containing 2% Fetal Clone II and 100 units/mL penicillin and 100 μg/mL streptomycin plus desired stimulus. Fresh medium without the stimulus served as control. After 24 hours, monocytes were collected by centrifugation and snap frozen at -80C for subsequent analysis. Conditioned media used as stimulus was removed from sub-confluent OPC tumor cell primary cultures and an equal volume added to monocytes for 24 hours. For co-culture experiments, monocytes were added to the same OPC tumor cells for 24 hours to determine if cell-cell contact was required for monocyte reprogramming.

PGE_2_ (Sigma-Aldrich, St. Louis, MO) was dissolved in 100% ETOH at 1mg/mL, and aliquots of 50 µg/mL prepared in E-media and stored at -80°C. Serial dilutions were prepared in E-media + 2% Fetal Clone II. Monocytes were stimulated as described above. Recombinant IL-1α and IL-1β (R & D Systems, Minneapolis, MN) were suspended in E-media + 2% Fetal Clone II at the desired concentrations and added to monocytes. Increasing concentrations of anakinra (Immunex, Thousand Oaks, CA) in E-media + 2% Fetal Clone II was added to freshly prepared monocytes for 1 hour prior to the addition of conditioned media or recombinant IL-1α. For the rilonacept experiments, increasing concentrations of rilonacept (Kiniksa Pharmaceuticals Ltd, London England) were added to the conditioned media for 1 hour at room temp prior to monocyte stimulation. For PGE_2_-pretreatment, control monocytes were cultured for 20 hrs. with 5,000 pg/mL PGE_2_ in E-media + 2% Fetal Clone II. Monocytes were then stimulated with an equal volume of conditioned media from SCC152, SCC-25, SCC-154, or with IL-1α in fresh medium at a final concentration of 200 pg/mL.

### Western blot of COX-2 protein

Stimulated monocytes from healthy donors were lysed in 1x TNE (Tris-EDTA-NP40) buffer and total protein was subject to western blot as previously described (20). Blots were probed overnight at 4°C, with antibodies against β-actin (Sigma-Aldrich, St. Louis, MO) and COX-2 (Cell Signaling Technologies, Danvers, MA). Membranes were scanned and quantified using the Sapphire Biomolecular Imager (Azure Biosystems, Dublin CA).

### RNA seq

Low passage OPC cell lines 2A3, SCC152, SCC154 and SCC25 were grown in E-media +10% Fetal Clone II as described above. Cells were adjusted to E media +2% Fetal Clone II for 48 hours, washed three times with PBS, trypsinized, pelleted at 500x g for 10 minutes at 4°C, washed once in cold PBS and re-pelleted. Total RNA was extracted using the RNeasy kit (Qiagen, Germantown MD) and quality controlled by Bioanalyzer RNA analysis (Agilent, Santa Clara CA). Sequencing libraries were prepared using the TruSeq stranded mRNA library prep kit and were sequenced with a NextSeq 500 (Illumina, San Diego CA). All RNAseq analysis was performed on Partek FLOW (Partek, Chesterfield MO). Briefly, high quality, trimmed sequences were aligned to the human genome (hg38) using STAR v2.7.8a. Sequences representing mapped genes were quantified and differential gene expression obtained by the DESeq2 method. Genes were considered differentially expressed if their false discovery rate adjusted t-test was p<0.05.

### Statistical analysis

Results are expressed as mean ± SD. Significance was determined by either a two-tailed unpaired Student t-test or by non-parametric Man-Whitney U test, as shown in the figure legends. p-values ≤0.05 were considered significant.

## Results

### Plasma levels of PGE_2_ are elevated in OPC patients and decrease post treatment

**Figure 1:**
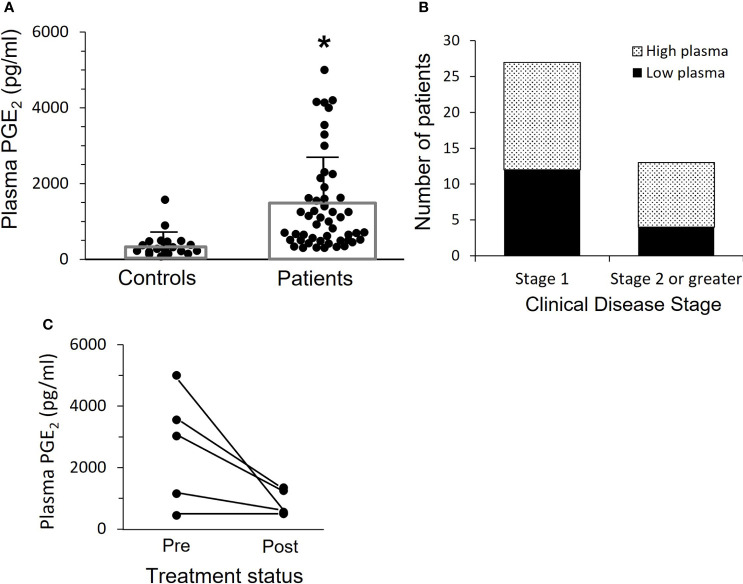
Patients with OPC have elevated levels of plasma PGE_2_. **(A)** PGE_2_ levels in plasma were measured by ELISA. Bars indicate mean ± SD. Plasma levels are significantly increased in patients, 1409 ± 169 pg/mL, n=52, as compared to controls, 372 ± 63 pg/mL, n=24, (*p < 0.0001, two-tailed unpaired t-test). **(B)** The patients shown in “A” with more advanced cancer (Stage 2 or greater) are more likely to have high plasma PGE_2_ levels, which we have set at 800 pg/ml. Results shown are not significant due to relatively small numbers, but clearly patients with more advanced disease are more likely to have higher plasma levels. **(C)** Plasma levels were measured a second time in five patients who were free of disease at least 16 months after surgery and subsequent treatment. Levels dropped in those with elevated PGE_2_ initially, and mean difference pre vs. post approached significance (p=0.06, paired t-test).

Plasma PGE_2_ levels for OPC patients are shown in **Figure 1A**. Plasma PGE_2_ levels were significantly elevated (p<0.0001) in patients (1409 +/- 169 pg/mL), compared to controls (372 +/- 63 pg/mL). Concentrations greater than 800 pg/ml were defined as high plasma levels. Elevated PGE_2_ levels were present in approximately 50% of the OPC patients. We asked whether there was a relationship between plasma level and clinical stage (**Figure 1B**). While not significant with these small numbers, there was a clear trend for patients with Stage 2 or greater disease to have elevated plasma levels.

Subsequent plasma samples were available more than 16 months after completion of treatment from 5 of the 52 OPC patients. We asked if plasma PGE_2_ levels were reduced when there was no evidence of disease (**Figure 1C**). There was a marked drop in mean PGE_2_ level from 2636 +/- 823 pg/mL at time of surgery to 830 +/- 193 pg/mL post-treatment in three patients who had elevated PGE_2_ expression and a smaller reduction in the PGE_2_ level in one patient who had a lower initial plasma PGE_2_ level, while the patient with an initial low plasma PGE_2_ level comparable to controls did not change over time.

### HPV16^+^ OPC patients have higher plasma PGE_2_ levels than HPV16^-^ OPC patients

**Figure 2:**
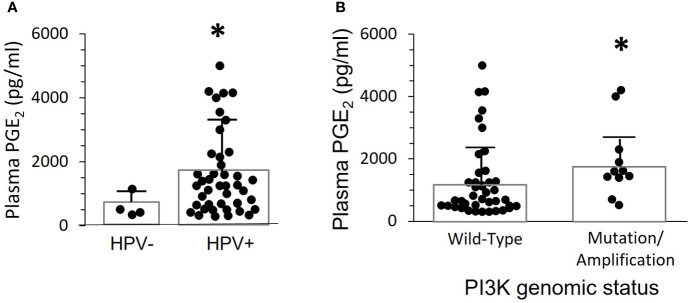
Plasma PGE_2_ levels correlate with the presence of HPV and gain of function mutations or amplification of *PIK3CA*. **(A)** PGE_2_ levels for the 52 patients in **Figure 1** were separated based on presence of HPV-16 in tumor biopsies. There was a significant difference between the two groups (*p=0.006, two-tailed unpaired t-test with Welch’s correction). **(B)** PGE_2_ levels were compared in 11 patients with either gene mutations or copy number amplifications, and 41 patients with normal sequence and copy-number. Bars indicate mean ± SD. Levels were significantly higher in the group with altered *PIK3CA*, 1919 ± 357 pg./mL, compared to wild-type, 1272 ± 188 pg/mL, (*p=0.014, Mann Whitney test).

Four of the 52 OPC patients studied for PGE_2_ plasma expression were HPV^-^. These patients had considerably lower levels of PGE_2_ (609 +/- 183 pg/mL) than the HPV16^+^ patients (1475 ± 180 pg/mL), (p=0.006) (**Figure 2A**). However, many HPV16^+^ OPC patients also had low PGE_2_ levels. Thus, HPV16 positivity is not sufficient to cause high plasma PGE_2_ expression.

### HPV16^+^ OPC patients with gain of function mutation/gene duplication in *PIK3CA*, the gene coding for Phosphoinositol-3-Kinase (PI3K), have elevated plasma PGE_2_ levels

Greater than 90% of *PIK3CA* activating mutations in OPC patients are in four “hotspots”, amino acids 542, 545, and 1047/1048 (21). In our patient population, one had a mutation at position 542, two had mutations at position 545, and one had a mutation at position 1047. There were also seven patients with gene copy number amplifications in the tumors, ranging from 1-3 extra copies per genome. None of the clinically normal oropharyngeal biopsies from these patients showed *PIK3CA* amplification or mutations. Plasma PGE_2_ levels were significantly higher (p=0.014) in patients with activating mutations or amplification (1919+/- 357 pg./mL) than in those with a wild-type *PIK3CA* genotype (1272 +/- 189 pg/mL) (**Figure 2B**). However, there were clearly patients with mutations or amplification who had low plasma PGE_2_ levels, and the highest level was in a patient without genetic alteration. Thus, elevated PI3K activity in the tumor is neither necessary nor sufficient to induce elevated PGE_2_ levels.

### *COX-2* mRNA expression is elevated in both tumors and PBMC of OPC patients

**Figure 3:**
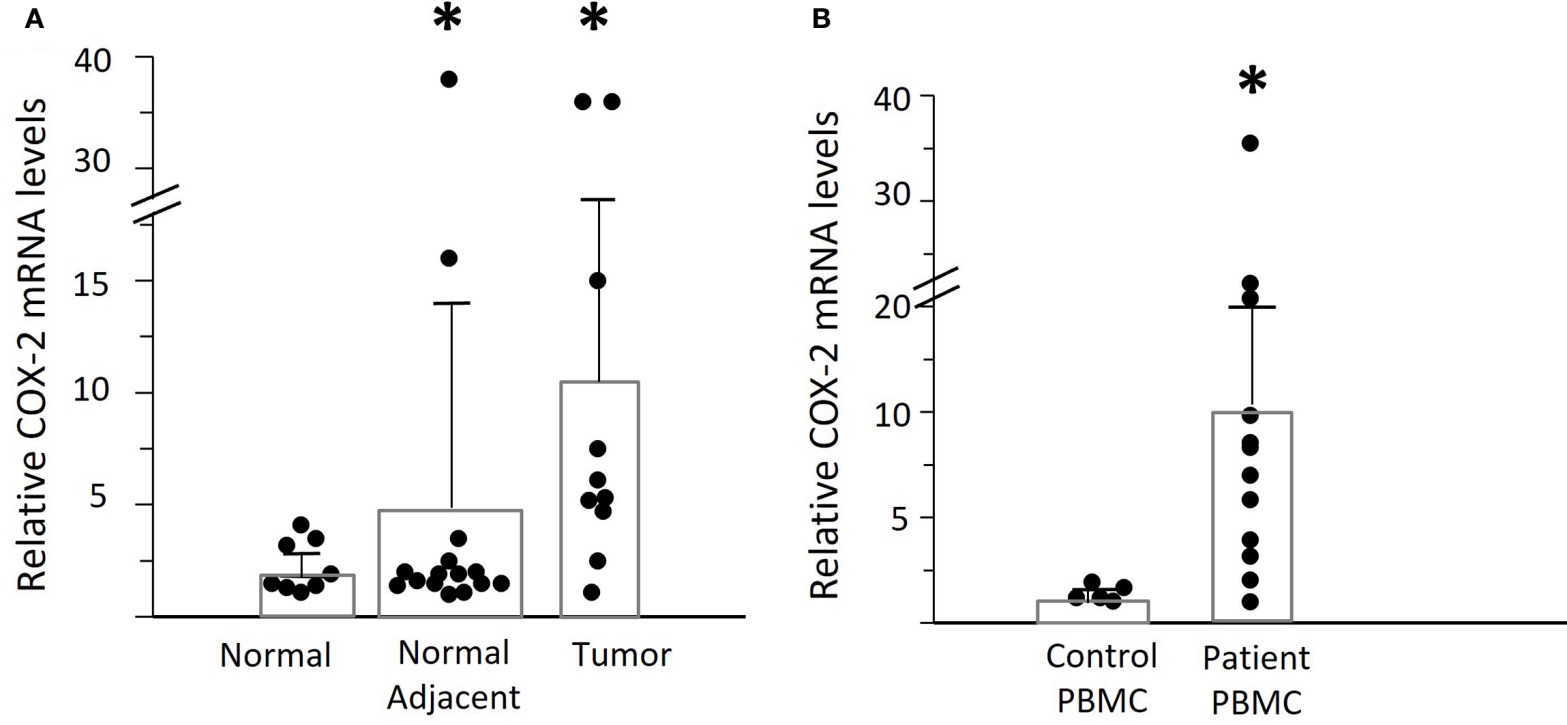
Expression of *COX-2* mRNA in OPC tissues and PBMC. **(A)** Levels of *COX-2* mRNA were assessed by q-RT-PCR in biopsies of OPC tissues (n=10) and clinically normal contralateral tissue (normal adjacent) from patients with OPC (n=15), relative to normal tonsil tissues (n=8.) Bars indicate mean +/- SD. There was a significant increase in expression in the tumor tissues of patients (*p=0.048, unpaired t test with Welch’s correction). **(B)** *COX-2* mRNA levels were measured in PBMC from patients with OPC (n=12) and expressed relative to the lowest measurable level in PBMC from controls (n=5). There was a statistically significant increase in the patients’ PBMC as compared to controls (*p<0.01, Mann-Whitney test).

We asked whether elevated tumor *COX-2* mRNA expression was the source of PGE_2_ in the plasma of OPC patients (**Figure 3A**). There was an 11-fold increase in the tumor tissues, compared to normal control tonsil from individuals without OPC. The contralateral “normal adjacent” tissues showed an intermediate 5-fold elevated expression. This could possibly reflect a response to elevated plasma PGE_2_ levels, since there is a positive feed-back loop with *COX-2* levels increasing in response to PGE_2_ stimulation in respiratory papilloma cells (22).

Despite the elevated *COX-2* levels in the tumors, we asked if that would be sufficient to account for the high plasma PGE_2_ levels in some patients, since the tumors are very small compared to the total volume of blood. We therefore measured the *COX-2* mRNA levels in PBMC from OPC patients compared to controls (**Figure 3B**). Mean levels of *COX-2* mRNA were much higher in patients than controls (patients: 10.87 ± 3.05, controls: 1.42 ± 0.17, p<0.01), but with high variability between patients. Monocytes were the predominant cell type expressing *COX-2*, and it was not detectable in NK cells, as anticipated (15). To better standardize the assays, all further experiments were therefore performed using purified monocytes

### Conditioned media from cultures of OPC tumors and tumor cell lines can induce *COX-2* mRNA in monocytes

**Figure 4:**
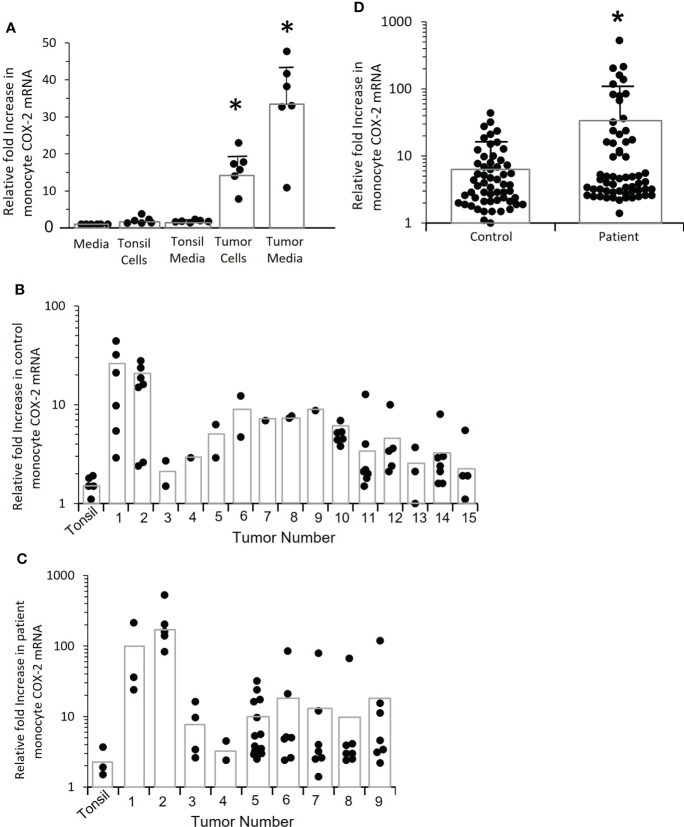
Tumor cells induce increased *COX-2* expression in control and patient-derived monocytes, patient monocytes are hyper-responsive. **(A)** Monocytes from controls were co-cultured with tumor cells derived from two OPC biopsies for 24 hrs. or incubated with conditioned medium obtained from the tumor cells, and *COX-2* mRNA measured by q-RT-PCR (*p=0.004). Normal tonsil keratinocytes and conditioned medium from tonsil cells served as controls. Results shown are relative to monocytes incubated with fresh media. Each dot within a given condition indicates a different preparation of monocytes (n=6), bars indicate mean ± SD. Both the tumor cells and the conditioned medium significantly increased *COX-2* mRNA (p=0.004, p=0.004 respectively, Mann-Whitney test). **(B)** Monocytes from controls were stimulated with conditioned media from 15 different tumor biopsy cultures or early passage tonsil keratinocytes (*p=0.002). Each dot within a given condition is a different preparation of monocytes. Bars indicate mean *COX-2* mRNA levels relative to monocytes treated with fresh medium (not shown). **(C)** OPC patient-derived monocytes were stimulated with conditioned medium from the first nine tumors shown in “B” or with tonsil-conditioned media. Each dot within a given condition is a different preparation of monocytes. Bars indicate mean *COX-2* mRNA levels relative to monocytes treated with fresh medium (not shown). **(D)** Data from tumor-conditioned media results in **(B, C)** was pooled for both control and patient monocytes and expressed relative to monocytes treated with fresh media (not shown). Bars indicate mean ± SD. There was a significantly increased response by patients’ monocytes compared to control monocytes (p=0.01, Mann-Whitney test).

To determine if OPC tumors drive monocyte *COX-2* expression, control monocytes were co-cultured with early passage cells from OPC biopsies from two patients or were treated with conditioned media from those tumors (**Figure 4A**). Cultures of tonsil epithelium or conditioned medium from tonsil cells served as a control. The tumors significantly induced monocyte *COX-2* expression (p=0.004), and the induction was mediated by a secreted factor and did not require cell-cell contact. The more robust induction with conditioned medium most likely reflects the fact that it was conditioned for 48 hours rather than the 24 hours used for tumor cell-monocyte co-cultures, thus increasing the accumulation of the inducing factor(s) in the medium. We then screened the ability of conditioned media from 15 separate tumors to stimulate control monocytes (**Figure 4B**), and screened nine of the tumors against patient-derived monocytes (**Figure 4C**). Although a few of the conditioned media were studied against a single control monocyte isolate, due to the limited amounts of conditioned medium, several initial conclusions can be drawn from this data. Tumors appeared to vary in their induction of *COX-2* mRNA. There was also marked variability in response to the same conditioned medium by monocytes from different individuals. Finally, the responses appeared to be greater with patients’ monocytes than with control monocytes. Combining responses to all the conditioned media by monocytes from patients and controls (**Figure 4D**) supported the conclusion that monocytes from OPC patients were hyper-responsive compared to controls (p=0.002).

### Conditioned media from oral/OPC cell lines increases *COX-2* mRNA levels in both control and OPC monocytes

**Figure 5:**
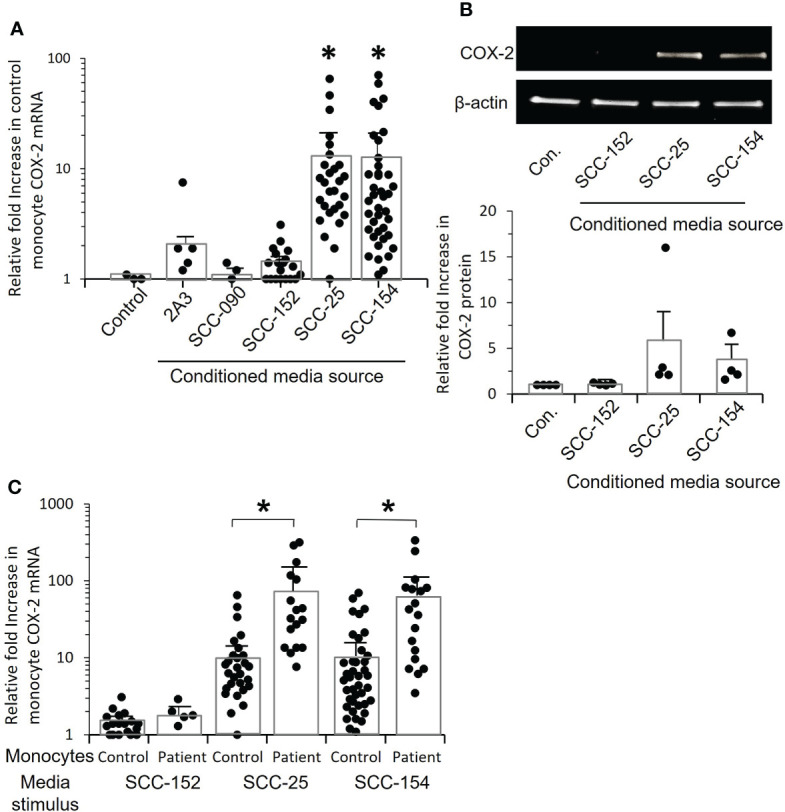
Conditioned media from tumor cell lines increase *COX-2* mRNA levels in control and OPC monocytes. **(A)** Monocytes from controls were stimulated for 24 hrs. with conditioned medium from five OPC/oral cancer cell lines, and *COX-2* mRNA levels measured by q-RT-PCR. Each dot within a given condition represents a different isolate of monocytes, bars indicate mean ± SD relative to media. There was a statistically significant increase in *COX-2* expression following stimulation with conditioned media from tumor cell lines SCC-25 (*p=0.008) and CC-154 (*p=0.008, Mann-Whitney test). **(B)** Monocytes from four controls were stimulated with conditioned medium from three of the cell lines, or with fresh media (con.) and cell extracts analyzed by western blot to confirm that COX-2 protein is also elevated. Results shown are one representative blot and the quantification of relative fold increase in COX-2 protein from the four experiments. **(C)** Monocytes from controls and OPC patients were stimulated with conditioned medium from SCC-152, SCC-25 and SCC-154, or incubated with fresh media (not shown) for 24 hrs. *COX-2* mRNA was measured by q-RT-PCR. Each dot within a given condition is a different isolate of monocytes. Bars indicate mean ± SD relative to fresh media. Expression was significantly increased in the patients’ monocytes compared to control monocytes when stimulated with conditioned media from SCC-25, (*p=0.003) and SCC-154, (*p=0.008, Mann-Whitney test) but not with conditioned medium from SCC-152.

To better understand the mechanism of *COX-2* induction, we needed a consistent source of conditioned medium. We screened several established oral/OPC cell lines and asked whether they induced *COX-2* mRNA in monocytes (**Figure 5**). Conditioned media from cell lines SCC-154 and SCC-25 significantly increased *COX-2* mRNA expression in control monocytes (p=0.017 and p=0.024 respectively) (**Figure 5A**). We confirmed by western blot that COX-2 protein was also increased, with the levels varying similarly to mRNA levels (**Figure 5B**).

*COX-2* mRNA expression was markedly increased in patients’ monocytes compared to control monocytes exposed to conditioned media from both SCC-154 (p=0.0003), and SCC-25 (p=0.0008), but not SCC-152 (**Figure 5C**). The ability to induce *COX-2* mRNA was not limited to HPV-positive tumors or cell lines, nor did the presence of HPV assure the *COX-2* induction phenotype. Tumor #5 shown in **Figure 4B**, and cell line SCC-25 are both HPV negative, while the other tumors and cell lines all contained and expressed HPV16.

### IL-1α, not PGE_2_, is a major inducing factor in conditioned medium

**Figure 6:**
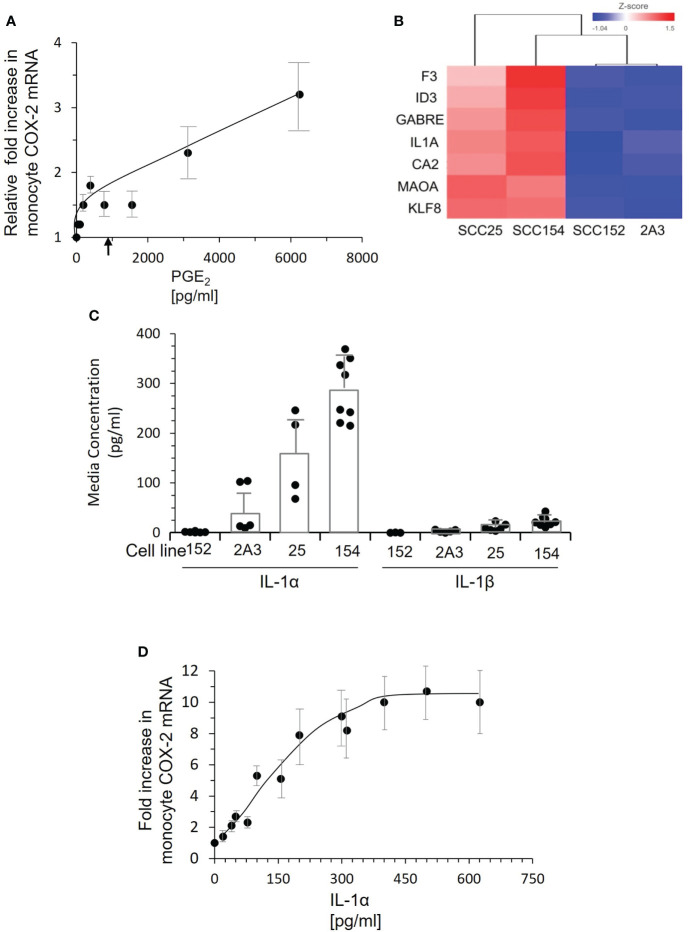
PGE_2_ is not the inducing factor. IL-1α present in conditioned medium of tumor lines SCC-25 and SCC-154 induces *COX-2* expression in monocytes. **(A)** Control monocytes were stimulated with increasing concentrations of PGE_2_, and *COX-2* mRNA measured by qPCR. Each dot represents the mean and SD of four measurements with different preparations of monocytes. There is a clear dose response, but the levels of COX-2 mRNA at doses below 2000 pg/mL are very low compared to the response of monocytes to conditioned media from tumor cells. The arrow indicates the mean level of PGE_2_ in conditioned medium from cultures of three tumor biopsies. **(B)** RNA-seq analysis of two OPC/oral cancer cell lines that induced *COX-2* compared to two cell lines that did not. The top genes selectively expressed by the inducing lines are shown. **(C)** Concentrations of IL-1α and IL-1β in conditioned medium from cell lines and primary OPC biopsy cultures were measured by ELISA. Dots represent different batches of media; bars are mean ± SD. **(D)** Control monocytes were stimulated with increasing concentrations of recombinant IL-1α for 18 hrs., and *COX-2* mRNA measured by q-RT PCR. Each dot is the mean ± SD of 3-4 measurements with separate isolates of monocytes. Approximately 50 pg/mL IL-1α is required to elicit a two-fold increase in *COX-2* mRNA, saturating at 300-400 pg/mL.

PGE_2_ stimulation can increase *COX-2* expression through a positive feed-back loop (23). We measured PGE_2_ levels in conditioned medium from tonsil keratinocytes, cells derived from three tumor biopsies, and cell lines SCC-25 and SCC-154. Tonsil-conditioned medium contained a mean of 34.6 ± 28 pg/mL PGE_2_ and the mean concentration in tumor-conditioned medium was 920.7 ± 240 pg/mL. PGE_2_ was undetectable in the cell line-conditioned medium. Control monocytes were then stimulated with increasing amounts of purified PGE_2_ added to fresh culture media (**Figure 6A**). While there was clearly a response, it was extremely small. At 6,000 pg/ml, the highest level measured in patient plasma, PGE_2_ induced a modest 3.2-fold increase in *COX-2* mRNA expression. At 1,000 pg/mL, the increase was less than two-fold. These results indicated that PGE_2_ is not the major factor inducing monocyte *COX-2.*

To identify the inducing factor(s) in OPC tumor conditioned media, we compared gene expression of the inducing cell lines SCC-25 and SCC-154 to lines 2A3 and SCC-152 by RNA-seq. Gene expression most significantly different in the two *COX-2* inducing cell lines is shown in **Figure 6B**. Of immediate interest was IL-1α, a cytokine that induces *COX-2* in tumor-associated fibroblasts (24). Thus, we measured IL-1α in conditioned medium from the cell lines (**Figure 6C**). SCC-25 and SCC-154 had much higher levels than SCC-152, which was higher than 2A3. Levels of IL-1β, which binds to the same receptor on cells, were extremely low in these conditioned medium. Stimulating control monocytes with increasing concentrations of recombinant IL-1α induced *COX-2* mRNA (**Figure 6D**). Approximately 250 pg/mL of IL-1α was required to approach the mean levels found in conditioned medium. Of note, none of the OPC patients’ plasma contained measurable levels of IL-1α, suggesting that monocyte exposure to IL-1α occurs locally *via* passage through the tumor or is delivered to blood monocytes by a different mechanism.

**Figure 7:**
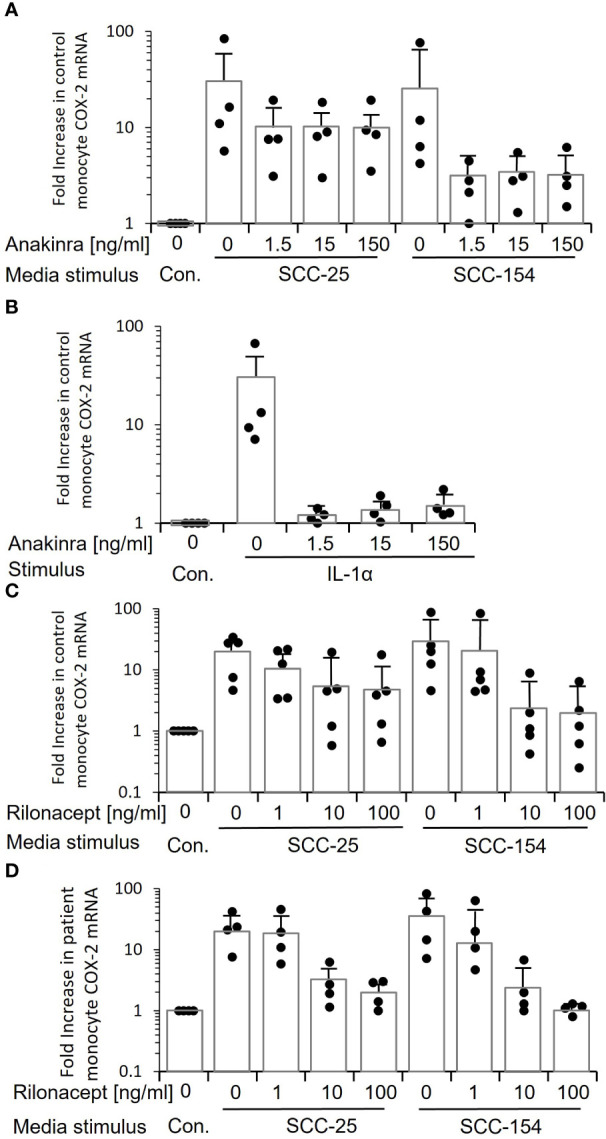
Inhibiting IL-1 signaling when stimulating monocytes with tumor line-conditioned medium reduces expression of *COX-2* but does not eliminate it. **(A)** Control monocytes from four individuals were treated with increasing concentrations of anakinra, a small molecule inhibitor of the IL-1 receptor, and then stimulated with conditioned medium from SCC-25 and SCC-154. *COX-2* mRNA was measured by q-RT PCR. Bars indicate mean ± SD relative to control fresh medium. Only a subset of monocytes showed a reduction in *COX-2* mRNA. **(B)** Control monocytes from four individuals were treated with anakinra as in “A”, then stimulated with increasing concentrations of recombinant IL-1α. Bars indicate mean ± SD relative to fresh medium (Con.) *COX-2* mRNA levels were reduced almost to control levels at all concentrations of anakinra, confirming that the inhibitor is effective and in excess. **(C)** Conditioned medium from SCC-25 and SCC-154 was pre-incubated with increasing concentrations of rilonacept, a recombinant IL-1 trap, and used to stimulate control monocytes from five individuals. *COX-2* mRNA was measured by q-RT PCR. Results are expressed relative to monocytes stimulated with fresh medium (Con.) Bars indicate mean ± SD. Only a subset of monocytes was completely blocked from responding. The highest level of *COX-2* mRNA expression at each concentration of rilonacept represents monocytes from the same donor. **(D)** Monocytes from four patients were stimulated with rilonacept-treated conditioned medium as described in “C”. Results are expressed relative to monocytes stimulated with fresh medium (Con). Bars indicate mean ± SD. Again, there was heterogeneity in response of monocytes to reduction in IL-1α stimulation although all patient-derived monocytes showed complete inhibition with the highest concentration of rilonacept-treated medium from SCC-154. Together, this data suggests that IL-1 is a major tumor paracrine factor inducing *COX-2* in monocytes, but monocytes from some individuals are responding to additional factors secreted from the tumor cells.

We treated control monocytes with three different doses of anakinra, a recombinant IL-1 receptor antagonist, and then stimulated them with conditioned medium from SCC-25 and SCC-154 (**Figure 7A**). All three doses reduced the induction of *COX-2* mRNA, but the reduction was not complete for monocytes from some individuals. Monocytes stimulated with conditioned medium from SCC-25 were less inhibited by anakinra than with SCC-154. Even the lowest dose of anakinra completely inhibited stimulation with recombinant IL-1α (**Figure 7B**), confirming that inhibition of the IL-1 receptor was complete.

Because small molecule inhibitors can have off-target effects, we also inhibited activation with rilonacept, a recombinant IL-1 receptor mimic that binds secreted IL-1, thus preventing it from binding to IL-1 receptors on cells (25). Pre-incubation of conditioned medium from SCC-25 and SCC-154 with increasing concentrations of rilonacept before stimulation significantly reduced *COX-2* mRNA induction in a dose-dependent manner (p<0.05) in both control monocytes (**Figure 7C**) and patient monocytes (**Figure 7D**). However, the reduction again was partial. Monocytes from some of the controls were relatively unchanged, especially with the SCC-25 conditioned medium, while others were completely inhibited. A similar pattern was seen with the patients’ monocytes, but all of them showed partial inhibition at 10ng/mL, and more so at 100 ng/mL. Thus, the data strongly indicates that while IL-1α is a primary inducer of monocyte *COX-2*, it is not the only paracrine factor(s) in the conditioned media and suggests that some patient and control monocytes are responding to the other factor(s).

**Figure 8:**
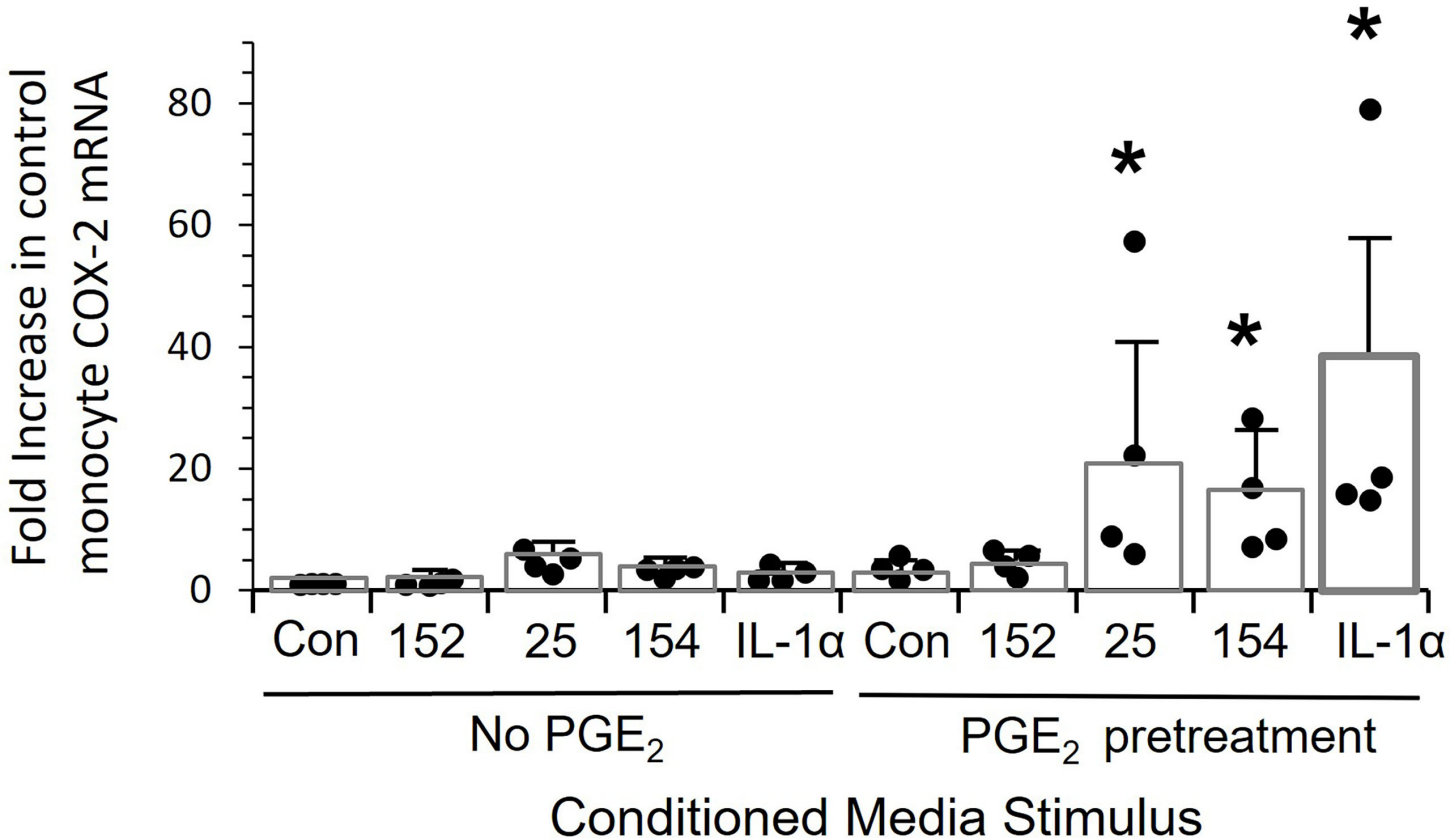
Pre-treatment of monocytes with PGE-2 increases the response to tumor line conditioned media and recombinant IL-1alpha. Control monocytes from four individuals were cultured for 20 hrs. ± 5,000 pg/mL PGE_2_, stimulated for 24 hrs with conditioned media from SCC152, SCC-25, SCC-154 or with 200 pg/mL IL-1α, and *COX-2* mRNA measured by RT-Q-PCR. Bars indicate mean +/- SD relative to untreated cells with no stimulation (Con.) Pre-treatment with PGE_2_ significantly increased response to conditioned media from SCC25 and SCC154 and recombinant IL-1α (p<0.03), but not the response to conditioned medium from SCC152. *p<0.03.

Finally, we asked the mechanism of hyper-responsiveness of patients’ monocytes. Pretreating control monocytes with PGE_2_ at a concentration found in OPC patients’ plasma significantly sensitized these cells to over-express *COX-2* (p<0.03) when treated with either conditioned media or IL-1α (**Figure 8**).

## Discussion

In this communication we began the characterization of the effect of OPC cells on monocyte function. We previous reported that systemic immune responses are disregulated in respiratory papillomas, a premalignant tumor caused by persistent HPV infection (9, 26–28). More recently, we reported that plasma levels of PGE_2_ are elevated in one third of respiratory papilloma patients (9). Here, we found elevated PGE_2_ plasma levels in approximately half of OPC patients, which is consistent with an earlier report for head and neck cancer patients in general, although the anatomic origin of those cancers is not described (29). This elevation reflects expression of *COX-2* by monocytes and is driven by factors secreted by the tumor cells. In our study all of the OPC patients’ tumors are from the palatine tonsil or base of tongue.

Recently, HPV16^+^ OPC patients have been divided by genetic expression into two groups: one with normal PI3K expression and the other with enhanced PI3K function (8). Here we further divide OPC patients by the functional ability of the tumor cells to up-regulate expression of *COX-2* in control and allogeneic OPC monocytes, independent of tumor PI3K gene status. Taken together, there are clearly more than two subdivisions of HPV16^+^ OPCs: some that show altered PI3K expression, others that do not, some that express a factor(s) that causes’ monocytes to up-regulate *COX-2*/PGE_2_, and others that do not. In addition, it appears that the genetic and functional differences of these OPC tumors are not linked, showing for the first time that up-regulation of PI3K by OPC tumors does not predict the ability of such tumors to induce enhanced monocyte *COX-2*/PGE_2_ overexpression. Thus, at least four subpopulations are present within the HPV16^+^ OPCs, and perhaps others may be identified based on the factors they express and the repertoire of immune-competent mediators that are expressed by monocytes stimulated by these factors. An interesting finding in our studies was noted using established oral/OPC cell lines. One HPV16^-^ cell line was able to induce monocytes to express *COX-2*/PGE_2_, while some HPV16^+^ cell lines could not. Thus, presence of HPV16 in the tumor appears to be neither necessary nor sufficient to induce gene expression changes in monocytes.

Recently, altered monocyte phenotypes in the blood of patients with different cancers were shown to serve as diagnostic (30, 31), predictive (32), and prognostic (33) biomarkers (34). Monocyte phenotypic alterations in cell surface marker, cytokine, and chemokine expression acquire reprograming during hematopoietic stem cell and blood monocyte development in patients with different cancers, recently reviewed by Olingy CE, et al. (35). The monocytes are also immunosuppressive (36). Transcriptional profiles of cancer-associated monocytes show little overlap between different cancer types and are markedly heterogenous within a given cancer type (30, 36–38). Immunosuppressive monocytes that emerge in cancer patients are associated with poor prognosis, thus implicating cancer-induced monocyte reprogramming as an important factor in tumor progression (39). Circulating classical monocytes infiltrate and replenish tissue macrophages and reprogramming of cancer-induced monocytes persists during their differentiation process into tissue macrophages, likely altering the function of their progeny in tissues (34). Our results shown in this communication suggest that monocytes in the blood of OPC patients can be reprogrammed, similar to the paradigm described in other cancer patients.

The induction of monocyte PGE_2_ by unknown factor(s) from adipose mesenchymal cells has been previously reported in an animal model of sepsis (40). This supports the concept that there is crosstalk between cells within tissues and circulating monocytes. However, little is known about the crosstalk between OPC cells and circulating monocytes. We have ruled out PGE_2_ itself as the potential factor (**Figure 6**) as exogenous PGE_2_ alone did not induce *COX-2* mRNA expression by control monocytes to the same extent as the conditioned medium, and PGE_2_ itself was not in high enough concentrations in tumor-conditioned media to be responsible for the levels of enhanced monocyte *COX-2* expression we observed. Rather, we have shown that IL-1α is a major inducer of monocyte *COX-2* by OPC cells. Moreover, control monocytes can be hypersensitized to express *COX-2* by pre-exposure to PGE_2_ and subsequently treating them with IL-α. This demonstrates that *COX-2* hyperexpression by control monocytes requires at least 2 different factors, at low concentrations found in the plasma or in conditioned media from OPC patients’ tumor cells. The mechanism of this effect is not known, but results are consistent with a possible upregulation of the IL-1 receptor.

The IL-1 family includes both pro-and anti-inflammatory cytokines. IL-1α and IL-1β are two of seven pro-inflammatory IL-1 mediators that can be pro- or anti-tumor in function. The pro-tumor and pro-metastasis effects of IL-1α have been well described in different malignancies (41–44), see recent review (45). IL-1α plays a role in crosstalk between immunocytes and tumors (44, 46), and it has been targeted in treating several cancers (47, 48). Notably, increased IL-1α production in tumors has been associated with increased metastases and poor survival of patients with head and neck squamous cell carcinomas (43). IL-1α signaling, leading to activation of NF-κB, is mediated by binding to its receptor, IL-1RI, followed by ligation of the co-receptor IL-1R3. The decoy receptor IL-1R2 competes for binding and inhibits IL-1α signaling (49–51). Differences in levels of expression of these receptors could contribute to the varied responses of control and patients’ monocytes to conditioned media from tumor cells as we have observed.

Reprograming of patients’ monocytes by factors secreted by the tumors could have both direct and indirect feedback effects on the tumors. Clearly, elevated expression of PGE_2_ and select cytokines/chemokines could impact the adaptive immune response, altering the ability of the immune system to control tumor growth and metastasis. Future studies will identify the repertoire of other immune mediators expressed by oropharyngeal cancers. Furthermore, characterization of the full repertoire of immune mediators expressed by control and patient monocytes in response to OPC is needed. These studies should reveal novel targets for future therapeutic intervention, help further subdivide the subpopulations of HPV16^+^ and HPV^-^ OPCs, correlation of these findings with clinical outcomes and more precisely predict appropriate treatment protocols. Finally, the markedly enhanced expression of *COX-2*/PGE_2_ by OPC patients’ monocytes needs further study to better understand the reciprocal crosstalk between OPC and the innate and adaptive immune systems.

## Data availability statement

The datasets presented in this study can be found in online repositories. The names of the repository/repositories and accession number(s) can be found below: GEO under accession ID GSE215973.

## Ethics statement

The studies involving human participants were reviewed and approved by Feinstein Institute Review Board. The patients/participants provided their written informed consent to participate in this study.

## Author contributions

JD, MI, FL and CP: Conceptualization, investigation, formal analysis, data curation, and writing, reviewing and editing. AA, DF, DK and LP: Conceptualization, obtaining patient tumor tissues and blood samples, and reviewing and editing. BS: Conceptualization, formal analysis, data curation, and writing, reviewing and editing. VB: Conceptualization, formal analysis, data curation, and writing, reviewing and editing, funding acquisition. All authors contributed to the article and approved the submitted version.

## Funding

This work was supported by the National Institute of Dental and Craniofacial Research (NIDCR) of the National Institutes of Health under Award Number DE017227 to Vincent Bonagura, and funding from the Feinstein Institutes for Medical Research.

## Conflict of interest

The authors declare that the research was conducted in the absence of any commercial or financial relationships that could be construed as a potential conflict of interest.

## Publisher’s note

All claims expressed in this article are solely those of the authors and do not necessarily represent those of their affiliated organizations, or those of the publisher, the editors and the reviewers. Any product that may be evaluated in this article, or claim that may be made by its manufacturer, is not guaranteed or endorsed by the publisher.
